# Preparation and Application of Cellulose-Based and Chitosan-Based Materials

**DOI:** 10.3390/polym18070812

**Published:** 2026-03-27

**Authors:** Guangmei Xia, Peng Jia

**Affiliations:** Key Laboratory of Pulp and Paper Science & Technology of Ministry of Education, State Key Laboratory of Green Papermaking and Resource Recycling, Faculty of Light Industry, Qilu University of Technology (Shandong Academy of Sciences), Jinan 250353, China

## 1. Introduction

The distinct inherent features of biomass resources include their carbon neutrality, noncompetition with food crops, abundant reserves, widespread distribution, low cost, and environmental friendliness [1,2,3]. The conversion of biomass into high-value functional materials, clean energy, and fine chemicals is a highly promising strategy to address pressing global challenges, including environmental degradation, energy scarcity, and climate change [4,5,6].

Among the diverse structural components of biomass, cellulose is the most abundant natural polymer on Earth, with its extensive sources including wood, agricultural straw, herbaceous plants, microorganisms, and even certain animal tissues. Cellulose can be processed into a wide variety of regenerated cellulose materials, such as cellulose films, cellulose fibers, cellulose sponges, and cellulose hydrogels [7,8,9]. Furthermore, targeted chemical modification of cellulose enables the synthesis of versatile cellulose derivatives, such as cellulose esters, cellulose ethers, and graft copolymers, which considerably broaden the application scope of cellulose to include numerous industrial and scientific fields [9,10,11].

Chitin is another critical natural polysaccharide that is extensively distributed in the exoskeletons of crustaceans (e.g., shrimps and crabs) as well as insects. Via deacetylation under alkaline conditions, chitin can be efficiently converted into chitosan. In contrast to insoluble chitin, chitosan exhibits solubility in dilute acidic aqueous solutions, which enhances its processability and facilitates subsequent material fabrication. Chitosan can be constructed into diverse functional materials, including films, fibers, separation membranes, tissue engineering scaffolds, and composite materials owing to its exceptional biodegradability, biocompatibility, film-forming capacity, and excellent adsorption performance. Chitosan thus demonstrates application potential in biomedical engineering, environmental remediation, food packaging, agricultural production, and other high-value sectors [12,13,14].

This Special Issue, entitled “Preparation and Application of Cellulose-Based and Chitosan-Based Materials”, focuses on cutting-edge research advances and novel findings related to cellulose- and chitosan-based materials, covering physical and chemical modification mechanisms, optimized preparation and processing technologies, performance enhancement strategies, and the expansion of novel application fields. This Special Issue collects eight high-quality original research papers, and a brief overview of their core content is provided below to arouse reader interest and encourage in-depth reading of these valuable studies.

## 2. Overview of Research Results

**Figure 1:**
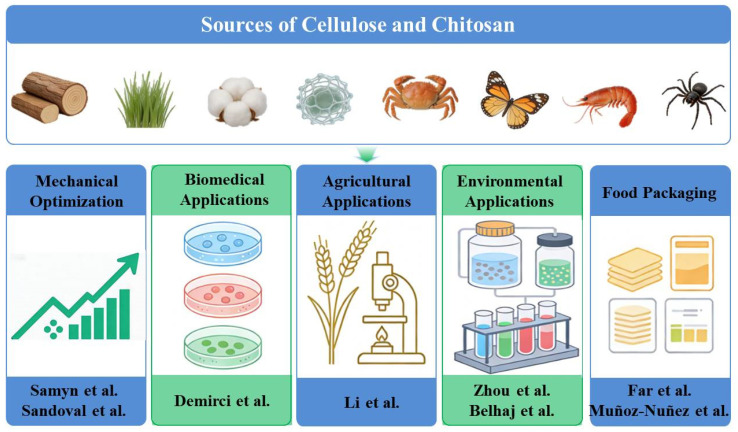
Schematic representation of the research topics covered by articles 1–8 published in this Special Issue, “Preparation and Application of Cellulose-Based and Chitosan-Based Materials”, based on the contributor list.

As illustrated in Figure 1, the eight articles published in this Special Issue are systematically categorized into five distinct groups based on the application fields of the developed cellulose- and chitosan-based materials, namely, the mechanical performance optimization of composites, the development of biomedical functional materials, agricultural biostimulants, environmental pollutant treatment, and food packaging material innovation. This classification demonstrates the vibrant research and broad application prospects in this interdisciplinary field.

### 2.1. Mechanical Performance Optimization of Cellulose-Based Composites

#### 2.1.1. Cellulose Acetate Microfiber Reinforced Thermoplastic Matrix

Sandoval et al. focused on the green enhancement in the mechanical properties of thermoplastic composites. Low-density polyethylene (LDPE) was selected as the polymer matrix, which was blended with potato starch (PS) to increase biodegradability. Cellulose acetate microfibers (MFCAs) were fabricated via electrospinning and incorporated into the matrix at three gradient loading levels of 0.05%, 0.15%, and 0.30%. The composite blends were processed via extrusion granulation and injection molding to prepare standard test specimens in accordance with ASTM D638 specifications.

The experimental results revealed that the cellulose acetate microfibers effectively reinforced the composite materials owing to their high aspect ratio and interfacial compatibility with the matrix. The tensile strength of the T3 group (84.7% LDPE, 15% PS) loaded with 0.30% MFCA reached 12.32 ± 0.5 MPa, representing an 11.79% increase compared with that of the T4 control group (85% LDPE, 15% PS), to which microfibers were not added. The melt flow index of the composite was precisely regulated, and the melt flow rate of the T2 group (84.85% LDPE, 15% PS) was close to that of pure LDPE, making the T2 group highly suitable for industrial extrusion processing. The study provides a feasible green modification scheme for thermoplastic matrices, and the as-prepared composites show potential for replacing partial synthetic polymers in industrial product manufacturing.

#### 2.1.2. Crosslinked Sprayed Cellulose Nanofibril Coatings

Samyn et al. addressed the critical limitation caused by the insufficient mechanical properties of pristine sprayed cellulose nanofibril (CNF) coatings, which restrict their practical industrial applications. Boric acid (BA) was employed as an efficient internal crosslinker, and polydopamine (PDA) served as an adhesion-promoting layer for fabricating two types of CNF-based coatings on glass substrates: multilayer structured coatings (CNF/BA) and homogeneous mixed coatings (CNF + BA).

The comprehensive performance of the coatings was evaluated via standardized tests including tape adhesion, rub resistance, cross-cut, and scratch resistance tests. The results demonstrated that the PDA/BA adhesion layer (with a BA concentration of 10 mM) endowed the coatings with high interfacial adhesion strength. The scratch resistance of the mixed coating (CNF + 10 mM BA) was 9 N, whereas that of the multilayer coating (CNF/100 mM BA) was 8 N, both of which were substantially higher than the scratch resistance of pristine CNF coatings (<0.5 N). After boric acid crosslinking, the coatings exhibited more stable water contact angles and reduced water penetration rates, alongside increased coating density and a slight decrease in optical transparency. The researchers effectively increased the mechanical durability of sprayed CNF coatings, enabling their practical application in industrial surface protection and high-contact surface protective coatings.

### 2.2. Chitosan-Based Materials in Biomedical Applications

Demirci aimed to overcome the core drawbacks of conventional chitosan surgical sutures, including their insufficient mechanical strength and single functional performance. Novel curcumin-loaded and TiO_2_-reinforced chitosan monofilament sutures were successfully fabricated via a dry jet-wet spinning technique.

The systematic characterization of the sutures indicated that adding 3% TiO_2_ significantly enhanced the tensile strength of chitosan sutures by 12.32%, reaching 189.41 MPa, while maintaining flexibility and ductility. After curcumin loading, the suture exhibited a free radical scavenging capacity of 43% at 125 h, far exceeding that of pristine chitosan sutures. The 24 h bacterial inactivation rate against *Staphylococcus aureus* was 98.87%, and the cumulative curcumin release rate was 77% within 25 h. Additionally, the suture induced the formation of a hydroxyapatite layer in simulated body fluid, demonstrating promising tissue regeneration potential. This multifunctional suture integrates wound suturing, antibacterial activity, antioxidant capacity, and tissue repair promotion features, indicating this suture is a competitive candidate for absorbable surgical sutures in clinical practice.

### 2.3. Chitooligosaccharides in Agricultural Applications

Li et al. focused on alleviating the adverse impacts of salt stress on agricultural crop production, a major constraint limiting global agricultural yield. Three deacetylases (NodB, VcCOD, and ArCE4A) were utilized to prepare three chitooligosaccharides (N-COS, C-COS, and A-COS) with distinct structural sequence arrangements using chitooligosaccharides with a polymerization degree of 2~6 as raw materials. Their regulatory effects on inducing salt resistance in wheat seedlings were systematically investigated.

All three chitooligosaccharides alleviated salt stress damage to varying degrees, and A-COS had the highest activity. When wheat seedlings under salt stress for 3 days were treated with 10 mg/L A-COS, the leaf length was restored, and the fresh and dry weights were restored by 20.40% and 6.64%, respectively. At the same time, 10 mg/L A-COS promoted proline accumulation and reduced the malondialdehyde content by 34.75%. Moreover, the Na^+^/K^+^ ratio was significantly reduced. As a result, the ion imbalance and oxidative damage caused by salt stress were effectively alleviated. The authors clarified the effect of the chitooligosaccharide sequence on the induction of plant salt resistance, providing a theoretical basis for the development of high-efficiency chitooligosaccharide-based plant biostimulants.

### 2.4. Cellulose- and Chitosan-Based Materials in Environmental Applications

#### 2.4.1. Phosphonated Chitosan for Separating Rare Earth Ions 

Zhou et al. targeted key challenges: the difficulty of separating rare earth ions and the low stability of chelating agents under acidic conditions, which hinder the efficient recovery of rare earth resources. In the work, acidic phosphonic chitosan (aPCS) was successfully synthesized via chemically modifying chitosan through the Mannich reaction.

Density functional theory (DFT) calculations revealed that the phosphonic acid groups of aPCS serve as the preferred binding sites for rare earth ions by forming stable bidentate chelate complexes. Complexation–`ultrafiltration experiments demonstrated that, at pH = 5 and a P/RE molar ratio of 10, the rejection rate of aPCS for La(III) was 97%, far exceeding the 70% rejection rate of conventional phosphorylated chitosan (PCS). Across the acidic pH range of 3~7, aPCS consistently exhibited superior La(III) rejection performance than PCS. This functional material holds promise for the selective extraction and purification of rare earth ions in acidic aqueous solutions, providing a novel and efficient solution for sustainably recovering rare earth resources.

#### 2.4.2. Agricultural-Waste-Derived Cellulose/ZnO Composites for Dye Removal

Belhaj et al. utilized agricultural pruning waste (almond and fig tree branches) as a low-cost and sustainable raw material for cellulose extraction. Cellulose/ZnO composites were prepared via the in situ deposition of ZnO nanoparticles for treating textile dye wastewater.

The characterization results showed that the size of the ZnO nanoparticles in the composite ranged from 13 to 26 nm, which were significantly smaller than pure ZnO nanoparticles (43 ± 12 nm). The ZnO nanoparticles were uniformly dispersed on the cellulose matrix. The composite removed 65% of the methylene blue (cationic dye) within 20 min and 45% of the anionic dyes including methyl orange and bromophenol blue. After three repeated adsorption–photocatalysis cycles, the removal rate for bromophenol blue only decreased by 15–19%, confirming their high reusability and stability. The researchers achieved the high-value utilization of agricultural waste biomass, providing a sustainable dual-functional adsorption–photocatalytic material for efficient textile wastewater treatment.

### 2.5. Cellulose- and Chitosan-Based Materials in Food Packaging Applications

#### 2.5.1. Air-Assisted Sprayed Cellulose Acetate/Chitosan Films

Far et al. employed the air-assisted solution spraying (AASS) technique to fabricate cellulose acetate (CA)/chitosan (CS) composite films, which were highly flexible, and the addition of external plasticizers was not required.

The optimized composite films exhibited a Young’s modulus below 1 GPa, with tensile strength and elongation at break exceeding 19 MPa and 2%, respectively, meeting the mechanical requirements for food packaging materials. With increases in chitosan content, the water and oil contact angles of the films increased, and the water barrier performance significantly improved. When the chitosan content was 7.5%, the film exhibited strong antibacterial activity against *Escherichia coli*, and the bacterial colony count reduced by two orders of magnitude compared with that achieved with neat cellulose acetate films. Compared with the traditional solution casting method, the AASS process features a faster drying speed and lower energy consumption, making the process more environmentally friendly and industrially scalable. The as-prepared sustainable and functional composite films are highly suitable for eco-friendly food packaging applications.

#### 2.5.2. 1-Methylimidazolium-Chitosan-Modified Films for Antimicrobial and Antioxidant Packaging

Muñoz-Nuñez et al. synthesized 1-methylimidazolium-chitosan (CS-MeIm) via chemical modification and incorporated CS-MeIm into chitosan film matrices at a loading of 10 wt%. Additionally, 1 or 5 wt% chitin nanowhiskers (ChNws) were introduced as reinforcing agents to further optimize the film’s performance.

Antioxidant capacity of the modified film drastically increased from 3 μmol Trolox per gram of film to 15 μmol Trolox per gram of film. The bacterial inactivation rate against *Escherichia coli* was 99.999%, indicating a significant improvement in antibacterial performance. Meanwhile, the water vapor transmission rate of the film decreased, and the mechanical properties were effectively regulated. Particularly, Young’s modulus increased, while the elongation at break slightly decreased. In summary, this modified chitosan film can be used as a biodegradable active packaging material for food preservation, effectively extending the shelf life of perishable foods.

The eight high-quality research papers included in this Special Issue focus on the targeted modification, performance optimization, and diversifying the applications of cellulose- and chitosan-based materials, covering core practical demands across multiple key fields:

Mechanical performance optimization: Two effective strategies, namely, the cellulose microfiber reinforcement of thermoplastic matrices and boric acid crosslinking of cellulose nanofibril coatings, significantly increased the mechanical robustness of cellulose-based materials, expanding their industrial application scenarios and practical usability.

Biomedical engineering: The developed TiO_2_-reinforced and curcumin-loaded chitosan surgical sutures integrate mechanical properties and multifunctional biological activity, opening a new direction for innovation in absorbable clinical surgical sutures.

Agricultural science: The correlation between chitooligosaccharide’s structural sequence and plant salt resistance induction was elucidated, providing reliable theoretical support for the development of green and high-efficiency plant biostimulants to address soil salinization issues.

Environmental remediation: The synthesized acidic phosphonated chitosan enables the efficient separation of rare earth ions in acidic solutions, and agricultural waste-derived cellulose/ZnO composites show synergistic adsorption–photocatalysis effects in textile dye wastewater treatment, enabling resource recovery and environmental pollution control.

Food packaging: Two novel functional chitosan/cellulose films enable plasticizer-free flexible processing and enhanced antibacterial-antioxidant performance, providing feasible and sustainable solutions for the development of eco-friendly food packaging materials.

Collectively, the research findings presented in this Special Issue demonstrate the immense application potential and sustainable value of cellulose- and chitosan-based materials, laying theoretical and experimental foundations for subsequent in-depth research and industrial translation in this field. The growing global research and continuous academic contributions from scholars worldwide have highlighted the urgent need for a follow-up Special Issue in this field, which will be entitled Preparation and Application of Cellulose-Based and Chitosan-Based Materials II.

To conclude this Editorial, I would like to extend a sincere and warm invitation to all researchers dedicated to this dynamic field to contribute their innovative, cutting-edge findings and join us in further advancing the fundamental research and practical application of cellulose- and chitosan-based biomass materials, driving the sustainable development of this promising research domain.
